# Effect of Body Mass Index on Intraoperative Complications During Hepatic Resection for Malignancy

**DOI:** 10.1111/ans.70250

**Published:** 2025-07-11

**Authors:** Jack Menzie, Thomas Coates, Amos Liew, Vanisha Fernando, Anderson Cheong, Lulu Xiao, Nicholas King, Yigeng Li, Travis Ackermann, Mithra Sritharan, Daniel Croagh, Geraldine Ooi

**Affiliations:** ^1^ Department of General Surgery Monash Health Clayton Australia; ^2^ Department of Upper Gastrointestinal and Hepatobiliary Surgery Monash Health Clayton Australia; ^3^ School of Clinical Sciences at Monash University Clayton Australia

**Keywords:** hepatectomy, intraoperative complications, liver neoplasms, obesity, postoperative complications

## Abstract

**Background:**

Evidence shows that high body mass index (BMI) contributes to increased postoperative complications in gastrointestinal surgery and suggests that it may contribute to intraoperative adverse events. We primarily aimed to determine if high BMI results in increased intraoperative adverse events in liver resections using the ClassIntra classification.

**Methods:**

A retrospective audit of liver resections under a single adult Hepatobiliary unit was performed from February 2018 to October 2023. We compared intraoperative adverse events and postoperative complications between BMI groups (‘Normal/low’ BMI < 25, ‘Overweight’ BMI 25–30 and Obese > 30). Resections were divided by complexity into minor, intermediate and major resections by extent of liver resection.

**Results:**

One hundred and ninety‐nine patients were included in the analyses. Higher BMI was associated with a significantly greater proportion of intraoperative complications using the ClassIntra classification (*p* = 0.022). At least one intraoperative complication was sustained by 33.3% and 38.2% of overweight and obese patients, respectively, compared to 19.1% in normal/low weight individuals. There were no differences in other intraoperative or postoperative outcomes or complications with a higher BMI, including estimated blood loss, morbidity by Clavien–Dindo classification, 30‐day readmission or mortality. Multivariate analysis showed that BMI class and diabetes status were significantly related to higher ClassIntra complication level (*p* = 0.0086).

**Conclusion:**

Higher BMI is associated with increased rates of intraoperative adverse events during liver resection surgery, by measure of ClassIntra classification. Prospective standardised assessment of intraoperative complications is required to confirm these findings.

## Graphical abstract

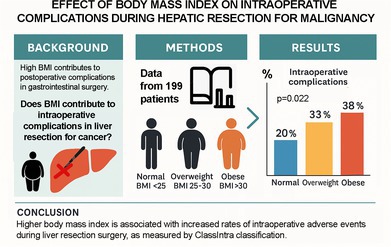

## 1 Introduction

Obesity rates have risen sharply in recent decades [1]. This is reflected in the cohort requiring hepatic cancer resections, with increasing concurrent obesity evident in this population [2]. Overweight and obesity, defined as a body mass index (BMI) over 25 and 30 kg/m^2^, respectively, are well‐established risk factors for perioperative complications and were once considered contraindications for certain operations due to technical challenges [3].

While advances in perioperative care and surgical techniques have reduced mortality and improved accessibility to surgical resection, postoperative complications, particularly with hepatectomies, remain high [4], although the literature is conflicting [5, 6]. Obesity additionally increases technical challenges, raising the likelihood of unplanned intraoperative events, such as conversion from laparoscopic to open surgery or increased blood loss [3, 6, 7]. Nonetheless, intraoperative adverse events associated with obesity in liver resections remain poorly studied [8].

In 2015, the CLASSification of Intraoperative Complications (ClassIntra version 1.0) was developed through a Delphi process with international experts, defining intraoperative adverse events as any deviation from the ideal surgical course (Table 1) [8]. This classification was validated in a prospective cohort study, demonstrating strong links between intraoperative events and postoperative outcomes across surgical disciplines [9]. Despite efforts to investigate the relationship between BMI and intraoperative adverse events [3, 10], a standardised grading system has not been utilised, and the impact of obesity on intraoperative complications in liver resections remains unclear.

**TABLE 1:** ClassIntra classification complications (adapted from ClassIntra version 1.0).

	Grade I	Grade II	Grade III	Grade IV
Treatment/intervention	None	Minor	Moderate	Major
Symptoms	Mild Asymptomatic	Moderate Not life threatening	Severe Potential life threatening	Life threatening/debilitating
Bleeding	Small‐calibre vessel Self‐limiting/manageable	Medium calibre artery or vein Ligation; use of tranexamic acid/ligation	Large calibre artery/vein with Hemodynamic instability Ligation or suture; blood transfusion	Life‐threatening Massive blood transfusion/ICU
Injury	Minimal organ lesion No treatment needed	Moderate organ lesion Requiring suture	Severe organ lesion Requiring minimal resection	Severe organ lesion Requiring major resection
Cautery	Small burn No treatment necessary	Moderate burn Noninvasive care	Severe burn Requiring surgical debridement	Life‐threatening burn ICU treatment
Arrhythmia	Without relevance, e.g., extrasystoles	Antiarrhythmic drugs No hemodynamic effect	Antiarrhythmic drug Hemodynamic effect	Electroconversion/defibrillation ± ICU admission

	Grade 0—Ideal intraoperative course		Grade V—Intraoperative death	
Examples from this study (haemostasis)	Minimal bleeding after parenchymal dissection, self‐limiting or managed with pressure	Bleeding requiring specific intervention after parenchymal dissection (clips, sutures, haemostatic agents)	EBL > 1000 mL, intraoperative blood transfusion, persistent bleeding after parenchymal dissection requiring multiple haemostatic strategies	Massive transfusion protocol

Abbreviation: EBL, estimated blood loss.

This study aims to provide insights into how obesity affects intraoperative and postoperative complications in liver resections for malignancy. We hypothesised that obesity would increase the risk of intraoperative and postoperative complications compared to nonobese patients, encompassing all types of liver resections from single segment resections to hepatectomies. We aim to identify areas of care affected by obesity and future areas of research to optimise care of patients undergoing liver resection.

## 2 Methods

We performed a retrospective review of all admissions for liver resections between February 2018 and October 2023 at Monash Health, Australia, to assess the impact of BMI on intraoperative adverse events and postoperative complications. Exemption from ethics approval was obtained, and the review was registered institutionally as a Quality Assurance activity.

Patients who underwent a liver resection were identified by ICD and MBS code from our electronic medical records and unit database. Inclusion criteria were: (1) age ≥ 18 years, (2) accurately recorded height and weight, (3) liver resection for malignancy, (4) confirmed or suspected malignant lesion. Exclusion criteria included: (1) age < 18 years, (2) septic or liver infections, such as abscess, (3) incomplete data.

Data collected included patient demographics like age, weight, surgical approach, American Society of Anaesthesiologists (ASA) score, neoadjuvant chemotherapy and radiotherapy. Operative records were analysed to determine the procedure, approach, estimated blood loss and operative time. Liver resections were grouped into three categories by extent of resection: (1) one to two wedge resections (‘minor’), (2) three or more wedge or sectionectomy (‘intermediate’) and (3) hemihepatectomy or extended hepatectomy, including resections requiring bile duct reconstruction (‘major’).

Two investigators independently reviewed these records to establish ClassIntra classification. Any disputes were resolved by consensus or a third reviewer when required. Use of the ClassIntra classification was discussed prior to grading. With regard to bleeding, examples of intraoperative complications by ClassIntra grades used in this study are given in Table 1. Additionally, after deliberation, conversion from laparoscopic to open surgery was not considered an intraoperative complication, as the initial laparoscopic approach to assess feasibility of laparoscopic resection is often used at our institution, with a controlled conversion to open procedure. Any intraoperative complications that led to conversion were documented. All intraoperative complications were recorded, with the highest grade for each patient being used for analysis.

Admission details were collected including length of stay, pre‐ and postoperative hemoglobin levels and blood transfusions. Postoperative complications classified by Clavien–Dindo classification, 30‐day readmission and 30‐day mortality were noted.

Patients were grouped according to BMI category, with a BMI of < 25 classified as normal/low, BMI 25–30 as overweight and BMI > 30 as obese.

The primary outcome was to compare ClassIntra grade of intraoperative complications between BMI groups. Secondary outcomes included intraoperative estimated blood loss, pringle manoeuvre time, postoperative complications as classified by the Clavien–Dindo classification [11], length of operation, length of stay and 30‐day outcomes.

Continuous nonparametric data were expressed as median values with ranges, while continuous parametric data was expressed as mean values with standard deviations, and categorical data as numbers with percentages. Nominal or ordinal outcomes were compared using Chi square or Fisher exact test and continuous variables by one‐way ANOVA as appropriate. Independent‐samples median test was used to compare median values. Pearson and Spearman correlation was used to calculate correlation between continuous and ordinal data, respectively. Multivariate analysis was conducted on R version 4.2.2 using both forward and backward stepwise selection of covariates. Clinically relevant demographic and operative variables, including age, gender and resection type, were included in the final model, while variables with negligible effect were excluded. Collinearity among covariates was assessed using variance inflation factors (VIFs) to ensure that multicollinearity did not influence the model. IBM SPSS statistics (version 30, IBM Corp, Armonk, NY) was used for statistical analysis, and a *p* < 0.05 was considered statistically significant.

## 3 Results

A total of 199 patients who underwent liver resection from February 2018 to October 2023 were identified. There were 68 (34.2%) normal/low weight patients, 63 (31.7%) overweight patients and 68 (34.2%) obese patients. No significant differences in baseline characteristics were found, including gender, ASA status and presence of diabetes (Table 2). There were 92 (46.2%) ‘minor’ resections, 46 (23.1%) ‘intermediate’ resections and 61 (30.7%) ‘major’ resections.

**TABLE 2:** Baseline characteristics (total population).

Baseline characteristics		Total (*n* = 199)	Normal/low BMI < 25 (*n* = 68)	Overweight BMI 25–30 (*n* = 63)	Obese BMI > 30 (*n* = 68)	*p*
BMI (kg/m^2^)		28 (5.9)	23 (1.9)	27 (1.4)	35 (4.6)	< 0.001
Age (years)		59.7 (3.2)	60.7 (11.5)	62.3 (14.2)*	56.8 (13.4)*	0.046
Gender (male)		117 (58.8%)	46 (67.6%)	37 (58.7%)	34 (50.0%)	0.112
Weight (kg)		79 (18.2)	63.3 (8.7)	75.9 (9.4)	97.5 (14.5)	< 0.001
ASA	1	9 (4.5%)	3 (4.4%)	4 (6.3%)	2 (2.9%)	0.937
	2	65 (32.7%)	21 (30.9%)	23 (36.5%)	21 (30.9%)	
	3	119 (59.8)	42 (61.8%)	34 (54.0%)	43 (63.2%)	
	4	6 (3.0%)	2 (2.9%)	2 (3.2%)	2 (2.9%)	
ECOG	0	177 (88.9%)	66 (92.6%)	57 (90.5%)	58 (85.3%)	0.321
	1	18 (9.1%)	4 (5.9%)	5 (6.3%)	9 (13.2%)	
	2	3 (1.5%)	1 (1.5%)	2 (3.2%)	0	
	3	1 (0.5%)	0	0	1 (1.5%)	
Pathology	CLM	90 (45.2%)	22 (32.4%)	37 (58.7%)	31 (45.6%)	0.071
	HCC	44 (22.1%)	22 (32.4%)	9 (14.3%)	13 (19.1%)	
	CCA	12 (6.0%)	5 (7.4%)	4 (6.3%)	3 (4.4%)	
	Gallbladder	12 (6.0%)	2 (2.9%)	4 (6.3%)	6 (8.8%)	
	Other	41 (20.6%)	17 (25.0%)	9 (14.3%)	15 (22.1%)	
Child pugh	Noncirrhotic	172 (86.4%)	56 (82.3%)	57 (90.5%)	59 (86.7%)	0.147
	CPA	24 (12.1%)	11 (16.2%)	5 (7.9%)	8 (11.8%)	
	CPB	3 (1.5%)	1 (1.5%)	1 (1.6%)	1 (1.5%)	
Diabetes		42 (21.1%)	16 (23.5%)	12 (19.0%)	14 (20.6%)	0.814
Ischaemic heart disease		22 (11.1%)	6 (8.8%)	10 (15.9%)	6 (8.8%)	0.337
Smoking status	Nonsmoker	114 (57.3%)	41 (60.3%)	34 (54.0%)	39 (57.4%)	0.719
	Ex‐smoker	57 (28.6%)	17 (25.0%)	18 (28.6%)	22 (32.4%)	
	Current	28 (14.1%)	10 (14.7%)	11 (17.5%)	7 (10.3%)	
Alcohol use	None/rare	135 (68.2%)	45 (67.2%)	45 (71.4%)	45 (66.2%)	0.963
	Moderation	10 (5.1%)	3 (4.5%)	3 (4.8%)	4 (5.9%)	
	Excess	53 (26.8%)	19 (28.5%)	15 (23.8%)	19 (27.9%)	
Neoadjuvant therapy		50 (25.1%)	16 (23.5%)	18 (28.6%)	16 (23.5%)	0.738
Approach	Lap	94 (47.3%)	32 (47%)	30 (47.6%)	32 (47.1%)	0.992
	Lap‐open	16 (8%)	5 (7.4%)	6 (9.5%)	5 (7.3%)	
	Open	89 (44.7%)	31 (45.6%)	27 (42.9%)	31 (45.6%)	
Resection complexity	Minor	92 (46.2%)	40 (58.8%)	27 (42.9%)	25 (36.8%)	0.129
	Intermediate	46 (23.1)	12 (17.7%)	15 (23.8%)	19 (27.9%)	
	Major	61 (30.7%)	16 (23.5%)	21 (33.3%)	24 (35.3%)	

*Note:* Figures expressed in mean (SD) or number (%) unless otherwise specified.

Abbreviations: ASA, American Society of Anesthesiologists classification; BMI, body mass index; CCA, cholangiocarcinoma; CLM, colorectal liver metastasis; CPA, Child Pugh A; CPB, Child Pugh B; ECOG, Eastern Cooperative Oncology Group performance status score; HCC, hepatocellular carcinoma.

* Post hoc test *p* < 0.05.

**FIGURE 1:**
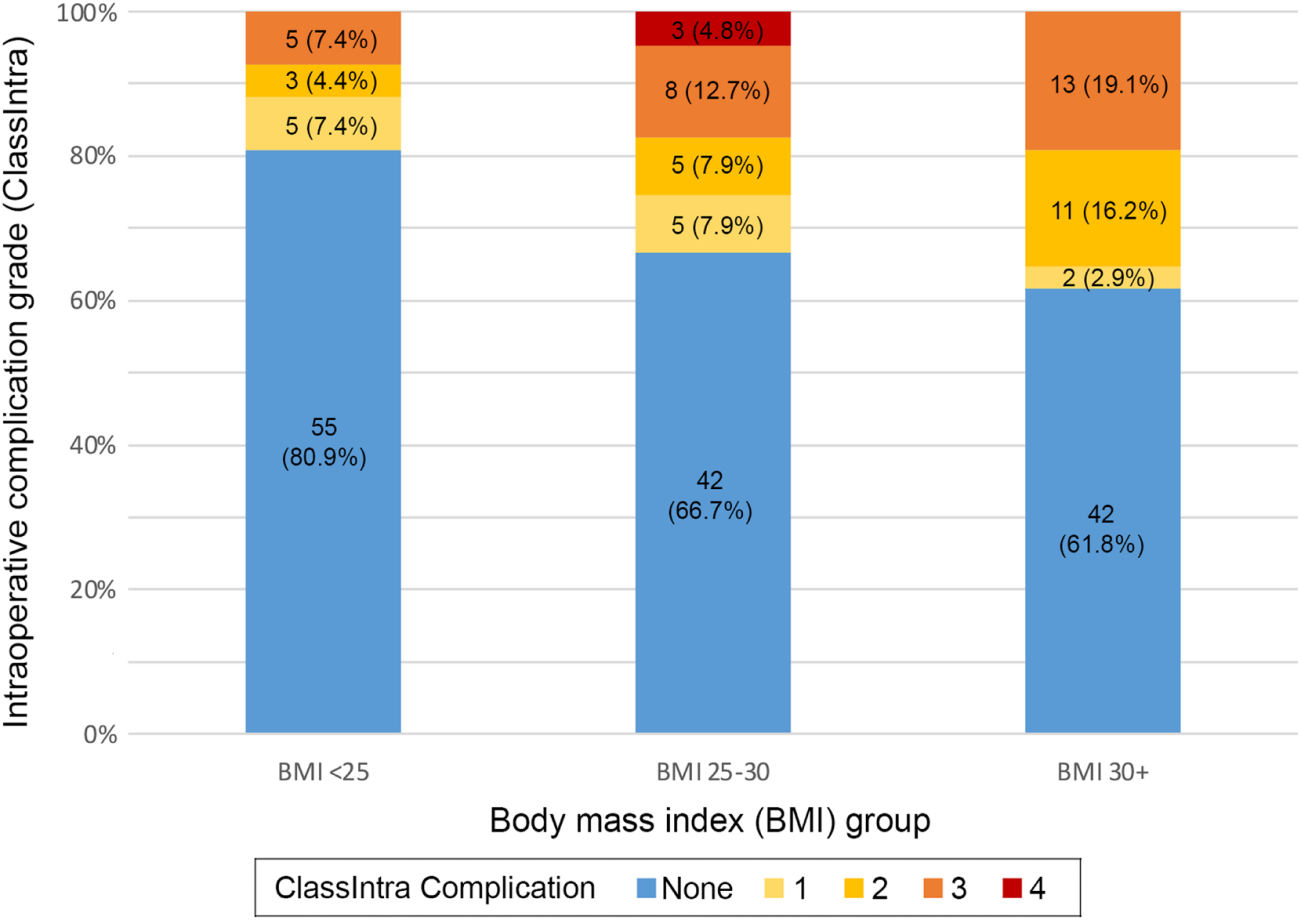
ClassIntra complications in all liver resections (minor, intermediate and major).

Sixty patients (30.2%) had at least one intraoperative complication that was recorded (Table 3); 12 (6%) had a ClassIntra Grade 1 complication, 19 (9.5%) had a ClassIntra Grade 2 and 29 (14.6%) had a ClassIntra Grade 3 or higher complication. Comparison of weight groups showed that there were higher rates of any intraoperative complications in the Overweight (*n* = 21, 33.3%) and Obese groups (*n* = 26, 38.2%) compared to the normal/low group (*n* = 13, 19.1%) (*p* = 0.042). The normal/low group showed much lower rates of ClassIntra 2 (*n* = 3, 4.4%) and ClassIntra ≥ 3 complications (*n* = 5, 7.4%). These findings were statistically significant (*p* = 0.022) (Table 3, Figure 1).

**TABLE 3:** Primary intraoperative and postoperative outcomes in all liver resections (total population).

Outcomes		Total (*n* = 199)	Normal/low BMI < 25 (*n* = 68)	Overweight BMI 25–30 (*n* = 63)	Obese BMI > 30 (*n* = 68)	*p*
Any intraoperative complication		60 (30.2%)	13 (19.1%)	21 (33.3%)	26 (38.2%)	0.042
ClassIntra grade	0	139 (69.9%)	55 (80.9%)	42 (66.7%)	42 (61.8%)	0.022
	1	12 (6%)	5 (7.4%)	5 (7.9%)	2 (2.9%)	
	2	19 (9.5%)	3 (4.4%)	5 (7.9%)	11 (16.2%)	
	3	26 (13.1%)	5 (7.4%)	8 (12.7%)	13 (19.1%)	
	4	3 (1.5%)	0	3 (4.8%)	0	
Clavien–Dindo ≥ 3		25 (12.6%)	6 (8.8%)	10 (15.9%)	9 (13.2%)	0.811
Clavien–Dindo grade	0	97 (48.7%)	35 (51.5%)	30 (47.6%)	32 (47.1%)	0.699
	1	34 (17.1%)	14 (20.6%)	10 (15.9%)	10 (14.7%)	
	2	43 (21.6%)	13 (19.1%)	13 (20.6%)	17 (25.0%)	
	3a	9 (4.5%)	2 (2.9%)	4 (6.3%)	3 (4.4%)	
	3b	9 (4.5%)	1 (1.5%)	3 (4.8%)	5 (7.4%)	
	4a	2 (1.0%)	0	2 (3.2%)	0	
	4b	2 (1.0%)	1 (1.5%)	0	1 (1.5%)	
	5	3 (1.5%)	2 (2.9%)	1 (1.6%)	0	
Length of operation (minutes)		257 (119)	231 (128)	264 (131)	277 (92)	0.069
Length of stay (days)		9 (9)	8 (7)	9 (13)	8 (7)	0.791
Estimated blood loss (mL)		732 (779)	422 (434)	919 (743)	736 (647)	0.172
Hb drop (g/L)		13.8 (13.4)	13.1 (2.8)	15.5 (16)	12.8 (11.2)	0.471
Bloods transfusion required?		27 (13.6%)	9 (13.2%)	11 (17.5%)	7 (10.3%)	0.368
Pringle used?		32 (16.1%)	10 (14.7%)	10 (15.9%)	12 (17.6%)	0.895
Pringle time (minutes)		4 (13)	4 (11)	4 (12)	5 (15)	0.963
Readmission (30 day)		21 (10.6%)	6 (8.8%)	6 (9.5%)	9 (13.2%)	0.669
Mortality (30 day)		3 (1.5%)	2 (2.9%)	1 (1.6%)	0	0.535

*Note:* Figures expressed in mean (SD) or number (%) unless otherwise specified.

Abbreviations: BMI, body mass index; Hb, haemoglobin.

Multivariate analysis was performed to assess variables associated with the chance of a higher intraoperative complication grade (Table 4). This showed that BMI class was an independent predictor of intraoperative complications, with patients in a higher BMI class being 121% more likely to have a higher ClassIntra complication grade (*p* = 0.0086). Diabetes was associated with a 196% increased risk of having a higher ClassIntra complication. An open procedure was protective, decreasing the chances of a higher intraoperative complication by 67.3% (*p* = 0.0029). With multivariate analysis, there was no association between resection complexity, age or gender and intraoperative complications.

**TABLE 4:** Multivariate analysis assessing the relationship of variables with intraoperative complication grade (by ClassIntra classification) in all liver resections.

Variable	Exp (coef)	SD	*t* value	*p*
Age	1.006	1.014	1.531	0.6703
BMI group	2.213	1.353	13.843	0.0086
Gender	1.090	1.411	1.285	0.8021
Diabetes	2.960	1.497	14.704	0.0072
Ischaemic heart disease	0.409	1.824	0.226	0.1364
Resection complexity	1.250	1.327	2.199	0.4308
Open approach	0.327	1.456	0.051	0.0029
Laparoscopic converted to open procedure	2.013	1.723	3.621	0.1982

*Note:* Model created by forward and backward stepwise selection of covariates, important demographic and operative variables, with variables of negligible effect excluded.

Abbreviation: BMI, body mass index.

**FIGURE 2:**
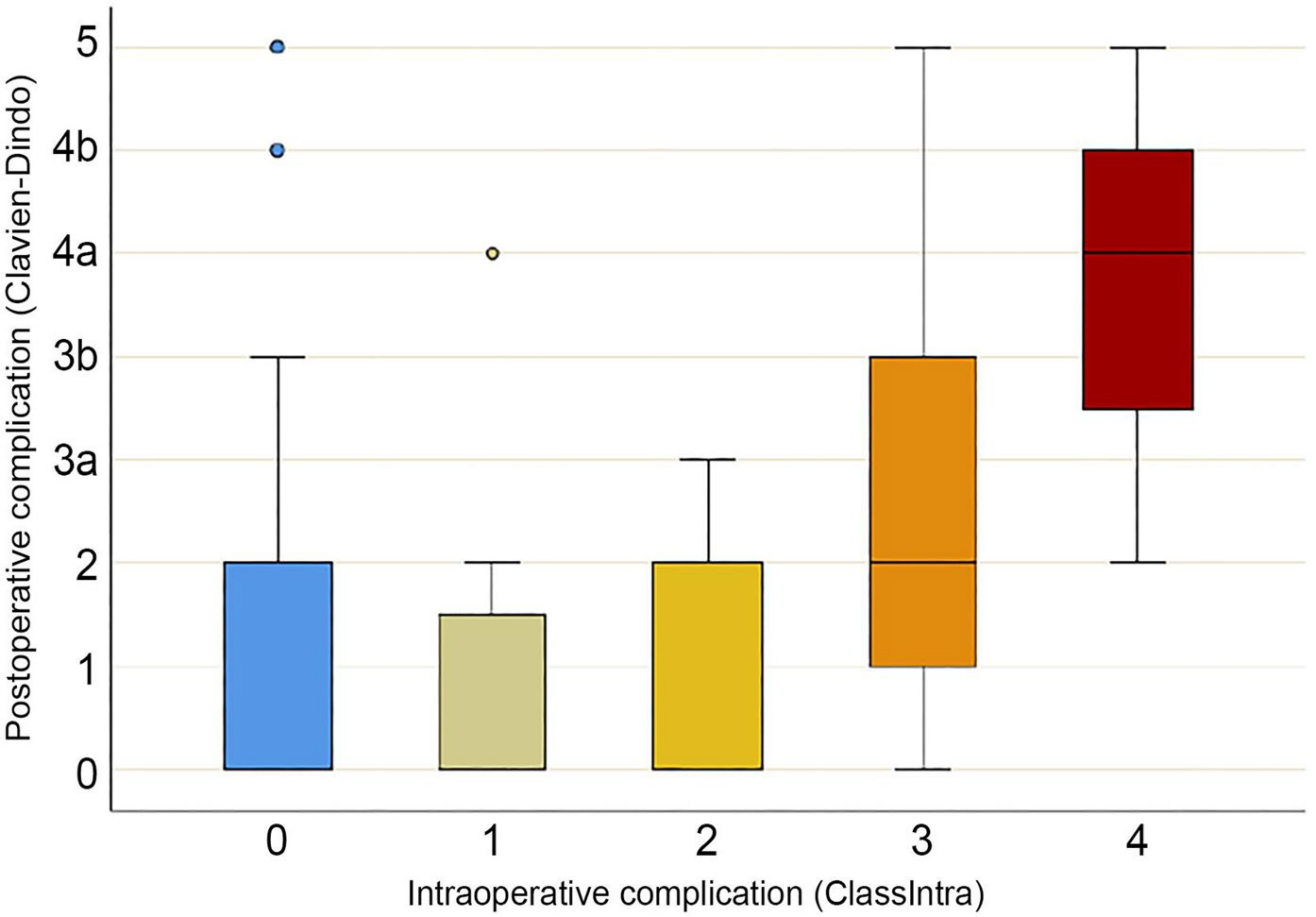
Relationship between occurrence and severity of intraoperative complications (as defined by the ClassIntra classification) and postoperative complications (as defined by the Clavien–Dindo classification).

There were no significant differences in other intraoperative measures or postoperative outcomes (Table 3). Despite this, the correlation of intraoperative with postoperative complications showed a significant relationship between the grade of ClassIntra and Clavien–Dindo complications (Spearman's *ρ* 0.265, *p* < 0.001) (Figure 2).

There were three 30‐day mortalities. Two patients suffered from haemorrhage, with one of these having intraoperative bleeding and vascular injury (ClassIntra 4), and the other had a delayed haemorrhage 5 days postresection with subsequent hypoxic brain injury (ClassIntra 0). One suffered progressive liver failure postoperatively (ClassIntra 3—haemorrhage).

Subgroup analyses of operation complexity (minor, intermediate and major) are presented in Tables S1–S8. This shows no significant differences in ClassIntra complications, postoperative complications or other outcomes with BMI group.

## 4 Discussion

Our study examines the effect of increased BMI on intraoperative complications and complexity in liver resections, by using the ClassIntra classification to systematically measure adverse intraoperative events. Analysis demonstrated that a higher BMI class was independently associated with a 2.2‐fold chance of a higher ClassIntra complication (*p* = 0.0086). Higher BMI was not significantly associated with any other surrogate measure of intraoperative complexity, including estimated blood loss, Pringle manoeuvre or blood transfusion. There was, however, a nonsignificant trend toward a longer operative time for overweight and obese patients compared to the normal/low group. The effect of obesity on intraoperative complications in a combined cohort of hepatic and pancreatic resections was reported by Gawria et al. [9], but to our knowledge, has not previously been studied in a specific liver resection population.

Intraoperative complications were significantly correlated with the development of postoperative complications (Spearman's *ρ* 0.265, *p* < 0.001). This is similar to a previous study of the correlation between intraoperative complications and postoperative complications in liver resections using the original 2016 CLASSIC classification [12]. This gives weight to the importance of developing strategies to minimise intraoperative complications, to improve overall outcomes.

Despite this, our data failed to show a significant difference in postoperative complications between BMI groups in our data. The detrimental effects of higher BMI on postoperative complications have previously been reported, showing increased surgical site infections, blood transfusions and overall morbidity [2, 3, 4, 10, 13, 14]. Alternatively, some studies contradict these findings [7, 15, 16, 17]. Some differences in reported outcomes may be attributed to study population, surgical technique and outcome definitions [3, 15, 16]. Our conflicting results could be due to our smaller patient population and relatively lower rates of Clavien–Dindo grade ≥ 3 postoperative complications.

We also found a significant association with diabetes status and higher grades of intraoperative complication. This may be due to the effects and associations of diabetes on liver steatosis and fatty liver disease. Liver steatosis increases the size of the liver as well as the fragility of the tissue and may lead to greater rates of unexpected bleeding.

Currently, we do not have a detailed appreciation of the exact technical issues encountered with obesity. Possible explanations include the increased fragility and weight of tissues, including increased liver steatosis, increasing risk of injury and bleeding, as well as intraabdominal and visceral adiposity making surgical access more challenging [3, 14]. Further study to gain a deeper understanding of how complications occur could be useful. This could help develop preoperative and intraoperative strategies that mitigate the challenges associated with operating on obesity. With nearly two thirds of the population affected with obesity and overweight, technical training aimed at identifying and managing specific challenges could assist in improving outcomes for this population.

The important question is we can reduce the complexity that obesity brings to major operative surgery such as liver resections. It is unknown whether perioperative intentional weight‐loss would reduce obesity‐related challenges. Certainly, weight reduction occurs rapidly from the liver, in as little as 2 weeks with very low‐calorie diets [18]. However intentional weight loss in preoperative patients with cancer presents many complex issues, including nutritional and functional status of patients, despite their obesity, and in the setting of potential cancer‐driven deconditioning. Despite an objectively ‘obese’ BMI, patients may also present with sarcopaenic obesity. Physical and psychological tolerance to obesity interventions in the setting of cancer management is also poorly studied, and strategies to improve adherence and efficacy are not well developed.

The ClassIntra classification introduces a standardised means of reporting intraoperative events [12, 19], and is a useful clinical and research tool. Incorporation in clinical practice improves handover from intraoperative to postoperative settings, and systematic transfer of information. Although previous studies have shown reasonable interobserver variability [20], these definitions are not specific and currently require some subjective interpretation. As such, grading will likely vary between surgical specialties, institutions and individuals. For example, intervention for the postparenchymal transection surface in liver surgery will differ between individuals, and interpretation of what is ‘normal’ versus management of an adverse event will vary. Intraoperative haemorrhage is particularly associated with interobserver variability [21]. This is one of the limitations of universal application of this classification, and future development of more specific specialty‐tailored guidelines may improve consistency.

There are several limitations to discuss. First, our study is a single‐centre study with a relatively small sample size, therefore limiting our statistical power. Despite this, our data did show significant differences in BMI groups and will help to develop future research. Additionally, we retrospectively gathered intraoperative data based on a review of documentation. To mitigate this, two independent reviewers came to a consensus, with a third review to resolve any disparity. Briefings before and after the commencement of data collection aimed at standardising assessment and grading of complications. Furthermore, other measures of intraoperative complexity, including operative time, surgical approach, blood loss, Pringle maneuver and postoperative complications were collected to complement this data.

In conclusion, our findings illustrate that overweight and obesity are independently associated with increased intraoperative complications during liver resections. Future studies should confirm these findings with prospectively collected and standardised measures of intraoperative complications. Comprehensive studies of strategies aimed at preoperative optimisation and improved surgical technique for intraoperative management of obesity will help improve outcomes in this growing population. These findings also emphasize the importance of ongoing population‐wide intervention for prevention and treatment of obesity.

## Disclosure

The authors have nothing to report.

## Conflicts of Interest

The authors declare no conflicts of interest.

## Supporting information


**Table S1.** Intermediate and major liver resections.
**Table S2**. Intermediate and major liver resections—outcomes.
**Table S3**. Minor liver resections—baseline characteristics.
**Table S4**. Minor liver resections—outcomes.
**Table S5**. Intermediate liver resections—baseline characteristics.
**Table S6**. Intermediate liver resections—outcomes.
**Table S7**. Major liver resections—baseline characteristics.
**Table S8**. Major liver resections—outcomes.

## Data Availability

The data that support the findings of this study are available from Monash Health. Restrictions apply to the availability of these data, which were used under license for this study. Data are available from the author(s) with the permission of Monash Health.
