# Supplementary material for: Effect of Body Mass Index on Intraoperative Complications During Hepatic Resection for Malignancy
Source: ANZ J Surg. 2025 Jul 11;95(7-8):1462–9. doi: 10.1111/ans.70250 (PMC12413578; doi:10.1111/ans.70250)
Supplement: Supplementary file 1 — Table S1. Intermediate and major liver resections. Table S2. Intermediate and major liver resections—outcomes. Table S3. Minor liver resections—baseline characteristics. Table S4. Minor liver resections—outcomes. Table S5. Intermediate liver resections—baseline characteristics. Table S6. Intermediate liver resections—outcomes. Table S7. Major liver resections—baseline characteristics. Table S8. Major liver resections—outcomes. [file ANS-95-1462-s001.pdf]

Supporting Information

Effect of body mass index on intraoperative complications during hepatic resection for malignancy

Dr. Jack Menzie (BSci MD)[1], Dr. Thomas Coates (BSci (Hons) MD)[1], Dr. Amos Liew (MBBS MS)[1], Dr. Vanisha Fernando (MD)[1], Dr. Anderson Cheong (MBBS)[1], Dr. Lulu Xiao (MD)[1], Dr. Nicholas King (MD)[1], Dr. Yigeng Li (MD)[1], Mr. Travis Ackermann (MBBS FRACS)[1,2], Mr. Mithra Sritharan (MBBS FRACS)[1,2], Mr. Daniel Croagh (MBBS FRACS)[1,2,3], Dr. Geraldine Ooi (MBBS FRACS)[1,2,3]*

Supplementary Table 1: Intermediate and Major liver resections – Baseline characteristics

**Baseline characteristics**		**Total (n=107)**	**Normal/low BMI <25 (n=28)**	**Overweight BMI 25-30 (n=36)**	**Obese BMI >30 (n=43)**	**p-value**
**BMI (kg/m^2)**		29.1 (6.1)	22.5 (2.0)$^{+}$	27.2 (1.4)*	34.9 (4.8)$^{*+}$	**<0.001**
**Age (years)**		58.9 (13.2)	61.6 (11.9)	63.3 (12.3)	53.6 (13.1)	**0.002**
**Gender (male)**		63 (58.9%)	20 (71.4%)	23 (63.9%)	20 (46.5%)	0.086
**Weight (kg)**		81.8 (18.9)	63.9 (9.6)	75.7 (9.4)	98.5 (15.3)	**<0.001**
**ASA**	**1**	6 (5.6%)	2 (7.1%)	3 (8.3%)	1 (2.3%)	0.751
	**2**	39 (36.4%)	9 (32.1%)	12 (33.3%)	18 (41.9%)	
	**3**	59 (55.1%)	17 (60.7%)	19 (52.8%)	23 (53.5%)	
	**4**	3 (2.8%)	0	2 (5.6%)	1 (2.3%)	
**ECOG**	**0**	95 (88.8%)	25 (89.3%)	33 (91.7%)	37 (86.0%)	0.159
	**1**	10 (9.3%)	3 (10.7%)	1 (2.8%)	6 (14.0%)	
	**2**	2 (1.9%)	0	2 (5.6%)	0	
**Diabetes**		20 (18.7%)	7 (25.0%)	7 (19.4%)	6 (14.0%)	0.501
**Ischaemic heart disease**		11 (10.3%)	1 (3.6%)	6 (16.7%)	4 (9.3%)	0.241
**Smoking status**	**Non-smoker**	60 (56.1%)	14 (50.0%)	17 (47.2%)	29 (67.4%)	0.410
	**Ex-smoker**	33 (30.8%)	10 (35.7%)	13 (36.1%)	10 (23.3%)	
	**Current**	14 (13.1%)	4 (14.3%)	6 (16.7%)	4 (9.3%)	
**Alcohol use**	**None/rare**	75 (70.8%)	22 (81.5%)	24 (66.7%)	29 (67.4%)	0.356
	**Moderation**	5 (4.7%)	2 (7.4%)	1 (2.8%)	2 (4.7%)	
	**Excess**	26 (24.5%)	3 (11.1%)	11 (30.6%)	12 (27.9%)	
**Neoadjuvant therapy**		28 (26.2%)	5 (17.9%)	10 (27.8%)	13 (30.2%)	0.492
**Approach**	**Lap**	60 (56.1%)	14 (50.0%)	23 (63.9%)	23 (53.5%)	0.564
	**Lap-Open**	35 (32.7%)	11 (39.3%)	8 (22.2%)	16 (37.2%)	
	**Open**	12 (11.2%)	3 (10.7%)	5 (13.9%)	4 (9.3%)	

Figures expressed in mean (SD) or number (%) unless otherwise specified. $^{*+}$*post-hoc test* $p<0.05$*. ASA – American Society of Anesthesiologist classification; BMI – Body mass index; ECOG – Eastern Cooperative Oncology Group performance status score.*

Supplementary Table 2: Intermediate and major liver resections - Outcomes

Outcomes		Total (n= 199)	Normal/low BMI<25 (n=68)	Overweight BMI 25-30 (n=63)	Obese BMI >30 (n=68)	p-value
Any intraoperative complication		38 (35.5%)	6 (21.4%)	16 (44.4%)	16 (37.2%)	0.155
ClassIntra grade	0	69(64.5%)	22(78.6%)	20(55.6%)	27(62.8%)	0.110
	1	4(3.7%)	1(3.6%)	3(8.3%)	0(0%)	
	2	11(10.3%)	1(3.6%)	3(8.3%)	7(16.3%)	
	3	20 (18.7%)	4 (14.3%)	7 (19.4%)	9 (20.9%)	
	4	3 (2.8%)	0	3 (8.3%)	0	
Clavien-Dindo ≥3		17(15.9%)	4(14.3%)	9(25%)	4(9.3%)	0.159
Clavien-Dindo grade	0	45 (42.1%)	13 (46.4%)	11 (30.6%)	21 (48.8%)	0.462
	1	20 (18.7%)	5 (17.9%)	6 (16.7%)	9 (20.9%)	
	2	25 (23.4%)	6 (21.4%)	10 (27.8%)	9 (20.9%)	
	3a	6 (5.6%)	2 (7.1%)	3 (8.3%)	1 (2.3%)	
	3b	5 (4.7%)	0	3 (8.3%)	2 (4.7%)	
	4a	2 (1.9%)	0	2 (5.6%)	0	
	4b	1 (0.9%)	0	0	1 (2.3%)	
	5	3 (2.8%)	2 (7.1%)	1 (2.8%)	0	
Length of operation (minutes)		293(124)	259(143)	317(133)	293(97)	0.175
Length of stay (days)		7(77)	7(29)	6(77)	6(33)	0.245
Estimated blood loss (ml)		638 (324)	456 (328)	765 (291)	575 (301)	0.051
Hb drop (g/L)		14.9 (13.7)	14.0 (9.1)	18.7 (17.8)	12.5 (11.8)	0.136
Bloods transfusion required?		22(20.6%)	6(21.4%)	10(27.7%)	6(14.0%)	0.321
Pringle used?		13 (12.1%)	4 (14.3%)	4 (11.1%)	5 (11.6%)	0.869
Pringle time (minutes)		30 (30)	30 (27)	22 (21)	40 (43)	0.772
Readmission (30 day)		9 (8.4%)	2 (7.1%)	3 (8.3%)	4 (9.3%)	1.000
Mortality (30 day)		3 (2.8%)	2 (7.1%)	1 (2.8%)	0	0.183

Figures expressed in mean (SD) or number (%) unless otherwise specified.

Supplementary Table 3: Minor Liver Resections – Baseline characteristics

Baseline characteristics		Total (n=92)	Normal/low BMI <25 (n=49)	Overweight BMI 25-30 (n=27)	Obese BMI >30 (n=25)	p-value
**BMI (kg/m^2)**		27(5.5)	22.5(1.9)	27.4(1.3)	34(4.2)	**<0.001**
**Age (years)**		61(13.1)	60.1(11.3)	61(16.5)	62(12.2)	0.792
**Gender (male)**		54 (58.7%)	26 (65.0%)	14 (51.9%)	14 (56.0%)	0.535
**Weight (kg)**		75.7(16.8)	62.9(8.2)	76(9.4)	95.7(13.1)	**<0.001**
**ASA**	**1**	3(3.3%)	1(2.5%)	1(3.7%)	1(4%)	0.227
	**2**	26(28.3%)	12(30%)	11(40.7%)	3(12%)	
	**3**	60(65.2%)	25(62.5%)	15(55.6%)	20(80%)	
	**4**	3 (3.30%)	2(5%)	0(0%)	1(4%)	
**ECOG**	**0**	83(90.2%)	38(95%)	24(88.9%)	21(84%)	0.250
	**1**	7(7.6%)	1(2.5%)	3(11.1%	3(12%	
	**2**	1(1.1%)	1(2.5%)	0(0%)	0(0%)	
	**3**	1(1.1%)	0(0%)	0(0%)	1(4%)	
**Diabetes**		22 (23.9%)	9 (22.5%)	5 (18.5%)	8 (32.0%)	0.503
**Ischaemic heart disease**		11 (12.0%)	5 (12.5%)	4 (14.8%)	2 (8.0%)	0.849
**Smoking status**	**Non-smoker**	54 (58.7%)	27 (67.5%)	17 (63.0%)	10 (40.0%)	0.085
	**Ex-smoker**	24 (26.1%)	7 (17.5%)	5 (18.5%)	12 (48.0%)	
	**Current**	14 (15.2%)	6 (15.0%)	5 (18.5%)	3 (12.0%)	
**Alcohol use**	**None/rare**	60 (65.2%)	23 (57.5%)	21 (77.8%)	16 (64.0%)	0.180
	**Moderation**	5 (5.4%)	1 (2.5%)	2 (7.4%)	2 (8.0%)	
	**Excess**	27 (29.3%)	16 (40.0%)	4 (14.8%)	7 (28.0%)	
**Neoadjuvant therapy**		22 (23.9%)	11 (27.5%)	8 (29.6%)	3 (12.0%)	0.271
**Approach**	**Lap**	34(37%)	18(45%)	7(26.9%)	9(36%)	0.589
	**Lap-Open**	54(58.7%)	20(50%)	19(70.4%)	15(60%)	
	**Open**	4(4.3%)	2(5%)	1(3.7%)	1(4%)	

Figures expressed in mean (SD) or number (%) unless otherwise specified. ASA – American Society of Anesthesiologist classification; BMI – Body mass index; ECOG – Eastern Cooperative Oncology Group performance status score.

Supplementary Table 4: Minor liver resections – Outcomes

Outcomes		Total (n=92)	Normal/low BMI <25 (n=49)	Overweight BMI 25-30 (n=27)	Obese BMI >30 (n=25)	p-value
**Any intraoperative complication**		22 (23.9%)	7 (17.5%)	5 (18.5%)	10 (40.0%)	0.087
**ClassIntra grade**	**0**	70(76.1%)	33(82.5%)	22(81.5%)	15(60%)	0.260
	**1**	8(8.7%)	4(10%)	2(7.4%)	2(8%)	
	**2**	8(8.7%)	2(5%)	2(7.4%)	4(16%)	
	**3**	6(6.5%)	1(2.5%)	1(3.7%)	4(16%)	
**Clavien-Dindo ≥3**		8 (8.7%)	2 (5.0%)	1 (3.7%)	5 (20.0%)	0.074
**Clavien-Dindo grade**	**0**	52(56.5%)	22(55%)	19(70.4%)	11(44%)	0.044
	**1**	14(15.2%)	9(22.5%)	4(14.8%)	1(4%)	
	**2**	18(19.6%)	7(17.5%)	3(11.1%)	8(32%)	
	**3a**	3(3.3%)	0(0%)	1(3.7%)	2(8%)	
	**3b**	4(4.30%)	1(2.5%)	0(0%)	3(12%)	
	**4a**	0	0	0	0	
	**4b**	1(1.1%)	1(2.5%)	0(0%)	0(0%)	
**Length of operation (minutes)**		216(99)	212(15)	191(87)	250(77)	0.097
**Length of stay (days)**		7(6)	8(7)	5(3)	7(5)	0.063
**Estimated blood loss (ml)**		494 (555)	352 (328)	350 (355)	1017 (975)	0.190
**Hb drop (g/L)**		12.4(13)	12.6(14.9)	11.4(12.4)	13.4(10.4)	0.862
**Bloods transfusion required?**		5(5.4%)	3(7.5%)	1(3.7%)	1(4%)	0.751
**Pringle used?**		19(20.7%)	6(15.0%)	6(22.2%)	7(28.0%)	0.440
**Pringle time (minutes)**		5(11)	3(8)	6(14)	5(12)	0.492
**Readmission (30 day)**		12(13%)	4(10%)	3(11.1%)	5(20%)	0.453
**Mortality (30 day)**		0	0	0	0	-

Figures expressed in mean (SD) or number (%) unless otherwise specified.

Supplementary Table 5: Intermediate liver resections – Baseline characteristics

Baseline characteristics		Total (n=46)	Normal/low BMI <25 (n=12)	Overweight BMI 25-30 (n=15)	Obese BMI >30 (n=19)	p-value
**BMI (kg/m^2)**		28.7(5.1)	23.0(1.63)	26.9(1.6)	33.6(3.5)	**<0.001**
**Age (years)**		58.6(12.5)	61.4(11.9)	59.3(12.1)	56.3(13.4)	0.534
**Gender (male)**		31 (67.4%)	10 (83.3%)	8 (53.3%)	13 (68.4%)	0.274
**Weight (kg)**		82.7(18.5)	65.9(9.4)	75.8(11.5)	98.8(14.2)	**<0.001**
**ASA**	**1**	2(4.3%)	0	2(13.3%)	0	0.451
	**2**	16(34.8%)	5(41.7%)	5(33.3%)	6(31.6%)	
	**3**	25(54.3%)	7(58.3%)	6(40%)	12(63.2%)	
	**4**	3 (6.5%)	0	2(13.3%)	1(5.3%)	
**ECOG**	**0**	40(87%)	10(83.3%)	13(86.7%)	17(89.5%)	0.173
	**1**	4(8.7%)	2(16.7%)	0	2(10.5%)	
	**2**	2(4.3%)	0	2(13.3%)	0	
**Diabetes**		11 (23.9%)	4 (33.3%)	5 (26.7%)	3 (15.8%)	0.563
**Ischaemic heart disease**		7 (15.2%)	1 (8.3%)	3 (20.0%)	3 (15.8%)	0.875
**Smoking status**	**Non-smoker**	25 (54.3%)	5 (41.7%)	9 (60.0%)	11 (57.9%)	0.566
	**Ex-smoker**	12 (26.1%)	5 (41.7%)	2 (13.3%)	5 (26.3%)	
	**Current**	9 (19.6%)	2 (16.7%)	4 (26.7%)	3 (15.8%)	
**Alcohol use**	**None/rare**	32 (69.6%)	10 (83.3%)	12 (80.0%)	10 (52.6%)	0.053
	**Moderation**	1 (2.2%)	1 (8.3%)	0	0	
	**Excess**	13 (28.3%)	1 (8.3%)	3 (20.0%)	9 (47.4%)	
**Neoadjuvant therapy**		22 (23.9%)	11 (27.5%)	8 (29.6%)	3 (12.0%)	0.271
**Approach**	**Lap**	22(47.8%)	3(25%)	12(80%)	7(36.8%)	**0.003**
	**Lap-Open**	20(43.5%)	8(66.7%)	1(6.7%)	11(57.9%)	
	**Open**	4(8.7%)	1(8.3%)	2(13.3%)	1(5.3%)	

Figures expressed in mean (SD) or number (%) unless otherwise specified. ASA – American Society of Anesthesiologist classification; BMI – Body mass index; ECOG – Eastern Cooperative Oncology Group performance status score.

Supplementary Table 6 – Intermediate liver resections – Outcomes

Outcomes		Total (n=46)	Normal/low BMI <25 (n=12)	Overweight BMI 25-30 (n=15)	Obese BMI >30 (n=19)	p-value
**Any intraoperative complication**		14 (30.4%)	2 (16.7%)	6 (40.0%)	6 (31.6%)	0.400
**ClassIntra grade**	**0**	32(69.6%)	10(83.3%)	9(60%)	13(68.4%)	0.370
	**1**	2(4.3%)	0	2(13.3%)	0(0%)	
	**2**	3(6.5%)	1(8.3%)	0	2(10.5%)	
	**3**	9(19.6%)	1(8.3%)	4(26.7%)	4(21.10%)	
**Clavien-Dindo ≥3**		4 (8.7%)	1 (8.3%)	3 (20.0%)	0	0.114
**Clavien-Dindo grade**	**0**	25(54.3%)	7(58.3%)	5(33.3%)	13(68.4%)	0.593
	**1**	9(19.6%)	2(16.7%)	4(26.7%)	3(15.8%)	
	**2**	8(17.4%)	2(16.7%)	3(20%)	3(15.8%)	
	**3a**	2(4.3%)	1(8.3%)	1(6.7%)	0(0%)	
	**3b**	1(2.2%)	0	1(6.7%)	0(0%)	
	**4a**	1(2.2%)	0	1(6.7%)	0(0%)	
**Length of operation (minutes)**		246(127)	185(77)	291(166)	249(104)	0.096
**Length of stay (days)**		9(14)	5(3)	15(23)	7(5)	0.120
**Estimated blood loss (ml)**		604 (613)	358 (375)	908 (794)	487 (476)	0.299
**Hb drop (g/L)**		15.2(11.4)	16.3(9.6)	16.6(10.9)	13.7(13)	0.743
**Bloods transfusion required?**		6(13%)	1(8.3%)	3(20%)	2(10.5%)	0.629
**Pringle used?**		8(17.4%)	3(25%)	3(20%)	2(10.5%)	0.619
**Pringle time (minutes)**		6(19)	8(20)	4(12)	6(24)	0.885
**Readmission (30 day)**		3(6.5%)	1(8.3%)	0	2(10.5%)	0.606
**Mortality (30 day)**		0	0	0	0	-

Figures expressed in mean (SD) or number (%) unless otherwise specified.

Supplementary Table 7: Major liver resections – Baseline characteristics

**Baseline characteristics**		**Total (n=61)**	**Normal/low BMI <25 (n=16)**	**Overweight BMI 25-30 (n=21)**	**Obese BMI >30 (n=24)**	**p-value**
**BMI (kg/m^2)**		29.4(6.8)	22.1(2.2)	27.5(1.3)	35.9(5.5)	**<0.001**
**Age (years)**		59.2(13.8)	61.8(12.2)	66(12)	51.4(12.8)	**<0.001**
**Gender (male)**		32 (52.5%)	10 (62.5%)	15 (71.4%)	7 (29.2%)	**0.012**
**Weight (kg)**		81.1(19.3)	62.4(9.7)	75.7(7.9)	98.4(16.5)	**<0.001**
**ASA**	**1**	4(6.7%)	2(13.3%)	1(4.8%)	1(4.2%)	0.496
	**2**	23(38.3%)	4(26.7%)	7(33.3%)	12(50%)	
	**3**	33(55%)	9(60%)	13(61.9%)	11(45.8%)	
**ECOG**	**0**	55(90.2%)	15(93.8%)	20(95.2%)	20(83.3%)	0.486
	**1**	6(9.8%)	1(6.3%)	1(4.8%)	4(16.7%	
**Diabetes**		9 (14.8%)	3 (18.8%)	3 (14.3%)	3 (12.5%)	0.902
**Ischaemic heart disease**		4 (6.6%)	0	3 (14.3%)	1 (4.2%)	0.234
**Smoking status**	**Non-smoker**	35 (57.4%)	9 (56.3%)	8 (38.1%)	18 (75.0%)	0.119
	**Ex-smoker**	21 (34.4%)	5 (31.3%)	11 (52.4%)	5 (20.8%)	
	**Current**	5 (8.2%)	2 (12.5%)	2 (9.5%)	1 (4.2%)	
**Alcohol use**	**None/rare**	43 (71.7%)	12 (80.0%)	12 (57.1%)	19 (79.2%)	0.267
	**Moderation**	4 (6.7%)	1 (6.7%)	1 (4.8%)	2 (8.3%)	
	**Excess**	13 (21.7%)	2 (13.3%)	8 (38.1%)	3 (12.5%)	
**Neoadjuvant therapy**		14 (23.0%)	2 (12.5%)	5 (23.8%)	7 (29.2%)	0.511
**Approach**	**Lap**	38(62.3%)	11(68.8%)	11(52.4%)	16(66.7%)	0.839
	**Lap-Open**	15(24.6%)	3(18.8%)	7(33.3%)	5(20.8%)	
	**Open**	8(13.1%)	2(12.5%)	3(14.3%)	3(12.5%)	

Figures expressed in mean (SD) or number (%) unless otherwise specified. ASA – American Society of Anesthesiologist classification; BMI – Body mass index; ECOG – Eastern Cooperative Oncology Group performance status score.

Supplementary Table 8 – Major liver resections – Outcomes

Outcomes		Total (n=61)	Normal/low BMI <25 (n=16)	Overweight BMI 25-30 (n=21)	Obese BMI >30 (n=24)	p-value
**Any intraoperative complication**		24 (39.3%)	4 (25.0%)	10 (47.6%)	10 (41.7%)	0.365
**ClassIntra grade**	**0**	37(60.7%)	12(75%)	11(52.4%)	14(58.3%)	0.183
	**1**	2(3.3%)	1(6.3%)	1(4.8%)	0(0%)	
	**2**	8(13.1%)	0	3(14.3%)	5(20.8%)	
	**3**	11(18%)	3(18.8%)	3(14.3%)	5(20.8%)	
	**4**	3(4.9%)	0	3(14.3%)	0	
**Clavien-Dindo ≥3**		13 (21.3%)	3 (18.8%)	6 (28.6%)	4 (16.7%)	0.627
**Clavien-Dindo grade**	**0**	20(32.8%)	6(37.5%)	6(28.6%)	8(33.3%)	0.821
	**1**	11(18%)	3(18.8%)	2(9.5%)	6(25%)	
	**2**	17(27.9%)	4(25%)	7(33.3%)	6(25%)	
	**3a**	4(6.6%)	1(6.3%)	2(9.5%)	1(4.2%)	
	**3b**	4(6.6%)	0	2(9.5%)	2(8.3%)	
	**4a**	1(1.6%)	0	1(4.8%)	0	
	**4b**	1(1.6%)	0	0	1(4.2%)	
	**5**	3(4.9%)	2(12.5%)	1(4.8%)	0	
**Length of operation (minutes)**		328(110)	315(157)	337(103)	328(78)	0.842
**Length of stay (days)**		11(8)	13(8)	11(9)	10(9)	0.716
**Estimated blood loss (ml)**		1024 (972)	667 (737)	1235 (1128)	713 (572)	0.542
**Hb drop (g/L)**		14.6(15.4)	11.9(8.5)	20(21.3)	11.4(10.9)	0.142
**Bloods transfusion required?**		16(26.2%)	5(31.3%)	7(33.3%)	4(16.7%)	0.401
**Pringle used?**		5(8.2%)	1(6.3)	1(4.8%)	3(12.5%)	0.727
**Pringle time (minutes)**		2(7)	1(6)	1(7)	3(9)	0.659
**Readmission (30 day)**		6(9.8%)	1(6.3%)	3(14.3%)	2(8.3%)	0.752
**Mortality (30 day)**		3(4.9%)	2(12.5%)	1(4.8%)	0	0.179

Figures expressed in mean (SD) or number (%) unless otherwise specified.
